# Analysis of the influence of shear-tensile resistance and rock-breaking effect of cutting holes

**DOI:** 10.1038/s41598-024-55640-2

**Published:** 2024-02-28

**Authors:** Antong Wan, Tiejun Tao, Xingchao Tian, Caijin Xie, Xia Liu, Zhenhua Zhao, Houying Zhang

**Affiliations:** 1 College of Civil Engineering, Guizhou University, Guiyang, 550025 China; 2 College of Mining, Guizhou University, Guiyang, 550025 China; 3 Northwest Engineering Co., Ltd. of CCCC First Highway Engineering Co., Ltd., Xi’an, 710000 China; 4 Guizhou Datong Road and Bridge Engineering Construction Co., Ltd, Guiyang, 550008 China

**Keywords:** Large-cross-section tunnels, Cutting blasting, Stress wave propagation, Dynamic damage to surrounding rock, Petrology, Civil engineering

## Abstract

In the process of drilling and blasting construction of large-cross-section tunnels, the layout of wedge-shaped cutting holes has a great influence on the effect of blasting. In this study, theoretical analysis and numerical simulation were used to assess the effect of different forms of cutting hole placement on blasting effectiveness. First, the fissure-inducing angle was proposed, a three-dimensional model of wedge-shaped cutting considering the effect of shear-tensile resistance was established, and theoretical analyses of cutting holes with different cutting angles and fissure-inducing angles were carried out. Second, the parameters of the Riedel–Hiermaier–Thoma model were determined based on the experimental data, and verified. Third, three-dimensional numerical models were established, and analyze the influence of different forms of hole deployment on the blasting effect from the perspective of stress wave propagation and dynamic damage to the surrounding rock. Finally, based on the theoretical analysis and numerical simulation results, the wedge-shaped hollowing holes were re-designed, and 20 tunnel blasting tests were carried out using this deployment method for large-section tunnel blasting, which verified the feasibility of this deployment method. The results of the study show that for level III surrounding rock, the angle of wedge-shaped cutting holes should meet 68° ≤ θ ≤ 70° and 70° ≤ β ≤ 72°. This study provides a kind of refined and efficient blasting for the drilling and blasting excavation process of large section tunnels.

## Introduction

Rapid economic growth has led to a continuous increase in the demand for road transportation, and traditional small-cross-section tunnels have poorly met the growing demand for transportation^1,2^. Large-cross-section tunnels are often bored via wedge-shaped cutting hole blasting to improve boring efficiency, increase cycle footage and ensure molding^3–5^ However, wedge-shaped cutting blasting has strict requirements on the placement of gun-holes, and unreasonable placement of cutting holes results in incomplete throwing of the rock in the slot cavity and a high rate of large pieces and affects the effectiveness of the subsequent blasting and the cyclic footage^6^. Therefore, the study of wedge-shaped cutting holes for specific surrounding rock levels has important engineering value for large-cross-section tunnel blasting.

In recent years, many scholars have conducted a large number of studies on wedge-shaped cutting blasting and achieved fruitful academic results. For example, Dai and Du^7^ proposed a method for calculating wedge cutting blasting parameters such as the distance between holes, the spacing between the bottoms of the holes and the charge of the cutting holes. Wang et al.^8^ used a simplified mechanical model to study the mechanism of cavity formation. Cheng et al.^9^ investigated the effect of explosive diameter on the destruction range and cutting space and verified that increasing the hole diameter and explosive diameter can effectively improve the utilization rate of gun-holes through on-site tests. Ding et al.^10^ studied the mechanical mechanism of orifice blasting and conducted tests with different forms of cutting blasting and proposed that under the condition of a weak coal rock body, when the depth of the hole exceeds 1.8m, the utilization of rock folder is significantly increased. Pu et al.^11^ obtained the factors affecting the effect of wedge-shaped cutting blasting through model tests and pointed out that the cutting angle is the main factor affecting the blasting effect. Yang et al.^12^ studied the influence of wedge cutting hole cutting angle on the cutting effect based on the model test data and proposed the optimal cutting angle.

During on-site tests, the blasting process is complex and extremely fast, the interference generated by the surrounding environment is not easy to exclude, the test period is long, the research cost is high, and the theoretical calculations can only solve some simplified problems^13,14^. With the rapid development of computer numerical simulation technology, numerical simulation has become a common method to study tunnel blasting. Hu et al.^15^ demonstrated the effectiveness of wedge cutting blasting in blasting excavation by adding a rock damage criterion to the finite element analysis software ANSYS/LS-DYNA. Meng et al.^16^ established a mechanical model of large diameter cut straight hole cutting blasting and verified the feasibility by numerical simulation. Cheng et al.^17^ simulated the propagation of the stress wave of wedge cutting blasting with different explosive diameters and revealed the effect of the law of explosive diameter on the stress distribution characteristics.

Qi et al.^18^ proposed the deployment of cutting holes and high-energy holes to solve the problem of inefficient tunnel boring.

Liu et al.^19^ analyzed the influence of different cutting angles on the blasting effect over three angles of stress wave propagation, the dynamic damage of surrounding rock and the evolution of the slot cavity based on the theory of wedge cutting rock breaking; combined with use of the finite element analysis software ANSYS/LS-DYNA, they proposed that the optimal cutting angle of level III surrounding rock is 60°.

At present, many studies have been carried out on parameters such as wedge cutting angle in terms of theoretical analysis, modeling tests and numerical simulation, but the coupled effect of cutting angle and fissure-inducing angle (The angle between the line connecting the center of a single-row wedge-shaped cutting holes on the tunnel face and the horizontal plane) on the blasting effect has not been reported. On-site tests have shown that the angle of placement of the cutting holes has an important influence on the effectiveness of blasting. For this reason, this paper is based on the wedge cutting blasting rock breaking mechanism, combined with mechanical tests, numerical simulation and on-site tests, and puts forward the best fissure-inducing angle of wedge cutting for large-cross-section tunnels with III perimeter rock. Take Tongliang–Anyue Expressway in Bayue Mountain l as an example; according to the real parameters of the site rock, a three-dimensional numerical model of 60°–80° cutting angle and 70°–90° fissure-inducing angle was established to analyze the blasting effect under different combinations of cutting angle and fissure-inducing angle. In order to verify the feasibility and potential popularization of the cutting hole deployment method proposed in this paper, 10 tunnel blasting tests were carried out in the Bayue Mountain Tunnel and the Gonghe Village Tunnel. The results of this study are intended to provide a refined and efficient blasting method for similar projects.

## Theoretical analysis

### Determination of physical parameters of surrounding rock

**Figure 1:**
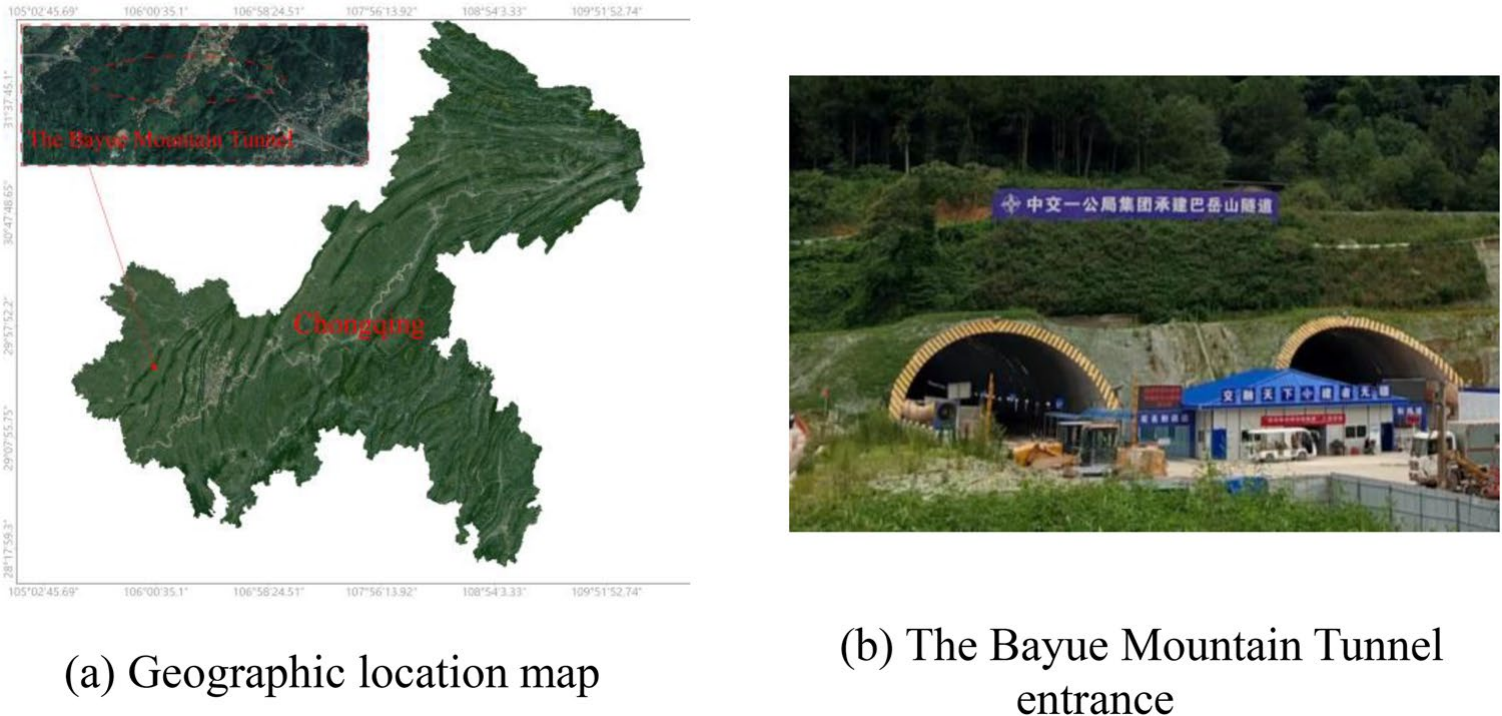
The Bayue mountain tunnel.

**Figure 2:**
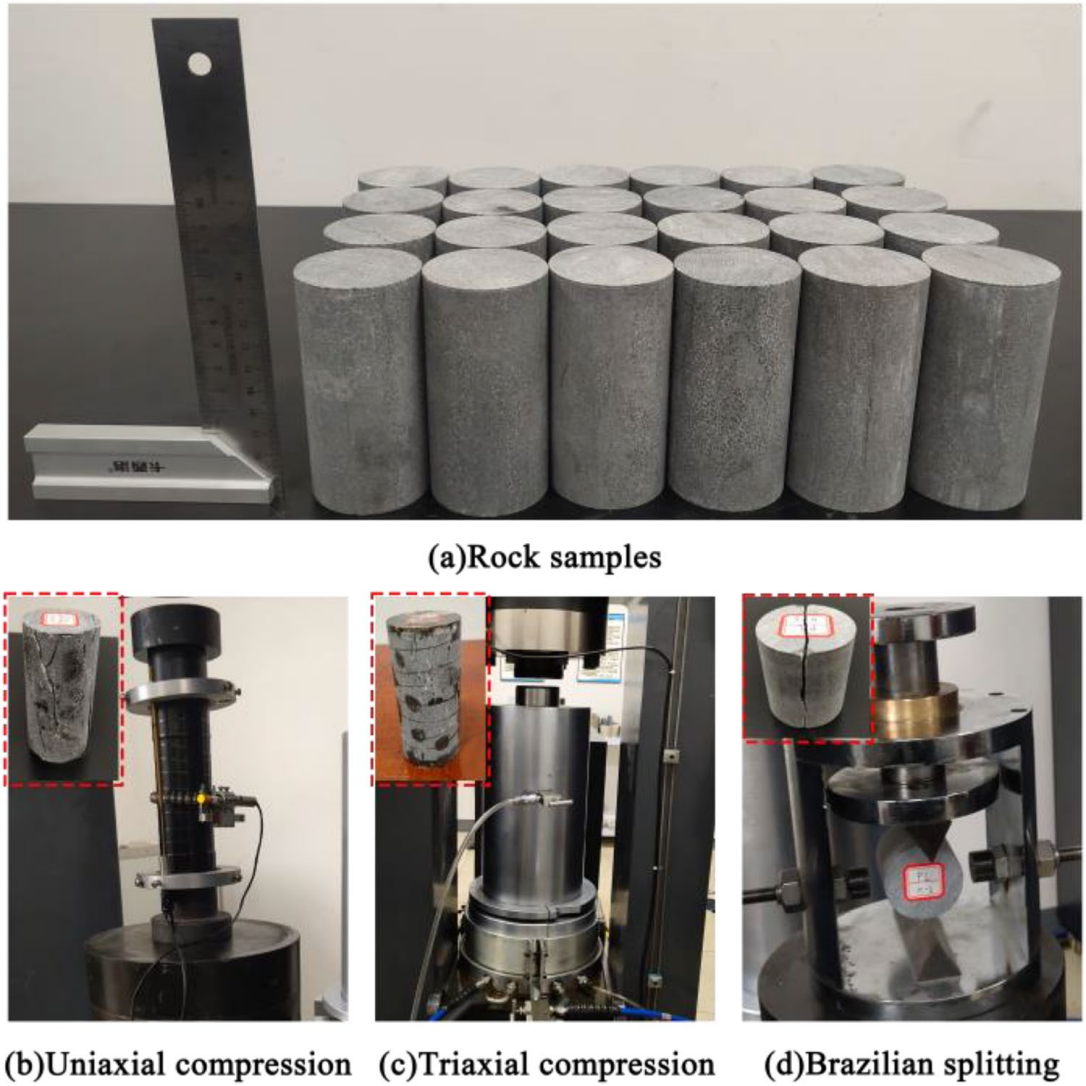
Static tests.

The Bayue Mountain Tunnel site area rocks are mainly limestone, and the surrounding rock level belongs to III, as shown in Fig. 1. Surrounding rock samples were taken from the construction site, and in order to avoid differences in the composition and structure of the rock samples, all test specimens were taken from the same rock mass, and according to the requirements of the International Society of Rock Mechanics^20^, the rock was made into standard specimens of 50 mm × 100 mm and 50 mm × 50 mm to carry out the uniaxial compression test, three-axial compression test and Brazilian cleavage test as shown in Fig. 2. The experimental results are shown in Tables 1 and 2.

**Table 1:** Physical and mechanical parameters of level III surrounding rock.

Surrounding rock level	Densities/(kg·m^−3^)	Uniaxial compressive strength/MPa	Tensile strength/MPa	Modulus of elasticity/GPa	longitudinal wave velocity/(m·s^−1^)	Poisson's ratio
III	2645	107.02	2.43	20.4	4153	0.28

**Table 2:** Parameters of limestone compressive strength under different confining pressure.

*σ*_2_ = *σ*_3_ (MPa)	*σ*_1_ (MPa)	*P*_0_*	*σ*_f_*
0	107.02	0.33	1.00
5	148.38	0.49	1.34
10	172.64	0.60	1.52
15	204.69	0.73	1.77
20	234.74	0.86	2.01
25	255.50	0.95	2.15
30	278.25	1.05	2.32
35	299.98	1.15	2.48
40	315.71	1.23	2.58

### Cutting angle and fissure-inducing angle determination method

Jun Dai^21^ proposed an expression for calculating the radius of the fracture zone:

1 $$R_{{\text{T}}} = R_{{\text{c}}} \left( {\frac{{\sigma_{{{\text{cd}}}} }}{{\sigma_{{{\text{td}}}} }}} \right)^{1/\beta } = r_{{\text{b}}} \left( {\frac{{P_{{\text{d}}} A}}{{\sqrt 2 \sigma_{{{\text{cd}}}} }}} \right)^{1/\alpha } \left( {\frac{{\sigma_{{{\text{cd}}}} }}{{\sigma_{{{\text{td}}}} }}} \right)^{1/\beta }$$

where $A = \left[ {(1 + \lambda )^{2} + \left( {1 + \lambda^{2} } \right) - 2\mu (1 - \mu )(1 - \lambda )^{2} } \right]^{1/2}$. *β* is the fissure zone attenuation index, *β* = 2−*μ*/(1−*μ*).

Existing studies have shown that, in order to ensure the effectiveness of cutting without causing damage to the preserved rock outside the tunnel design contour, the distance from the cutting hole to the tunnel design contour is greater than or equal to the radius of the fissure zone *R*_*T*_, and the spacing of the chambers at the bottom of the hollowing hole is less than or equal to two times the radius of the fissure zone *R*_*T*_^22,23^.

**Figure 3:**
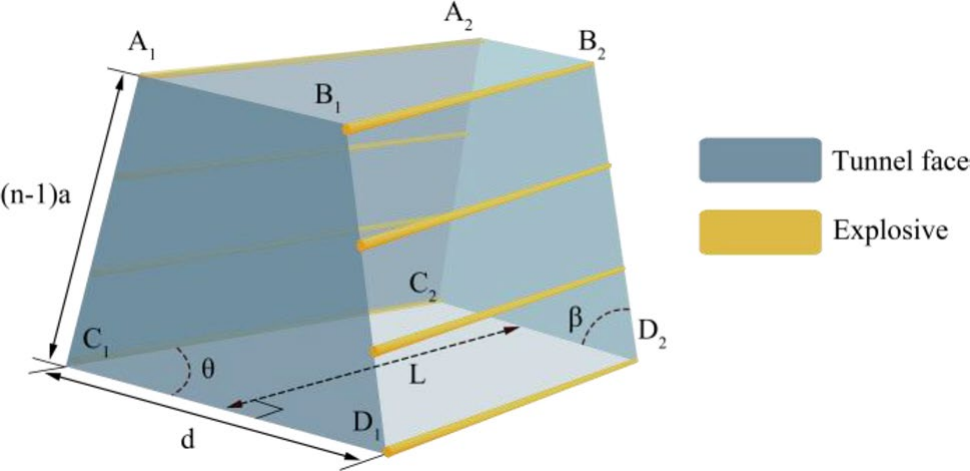
Three-dimensional model of wedge-shaped cutting.

Confining pressure damage mainly occurred in the form of shear action parallel to the free surface of the tensile damage when blasting action upon the rock, as a sum of the shear and tensile force, was greater than the ultimate shear and tensile strength of the rock, resulting in damage to the rock. Figure 3 is a three-dimensional model of wedge-shaped cutting, with face ABDC indicating the free surface, the red dotted line indicating the cutting holes, each cycle of the depth *L* = 3 m, the number of holes in each row *n* = 8 and the cutting holes spacing a = 0.4 m. In this paper, we chose No. 2 emulsion explosives for the calculation of the explosive density of *ρ*_*0*_ = 1240 kg·m^3^, with bursting speed *D* = 4200 m/s, the diameter of the holes *d*_*b*_ = 42 mm, the length of the charge of 2.4 m, and the length of the charge *R*_*T*_ = 2.8 m. In order to push the cutting holes outward as far as possible, the lower row of cutting holes cavity spacing *d* = 2* R*_*T*_ = 5.6 m.

The shear resistance of face A_1_B_1_B_2_A_2_ is:

2 $$Q_{{A_{1} B_{1} B_{2} A_{2} }} = ({\text{c}} + \sigma_{1} \tan \varphi )\left[ {d - 2(n - 1)a\cos \beta - \frac{L}{\tan \theta }} \right]L$$

where *c* is the cohesion of the rock. *φ* is the angle of internal friction of the rock. *σ*_*1*_ is the positive stress on the face A_1_B_1_B_2_A_2_, *σ*_*1*_ = γ*z*. γ is the rock mass. *z* is the distance of the face from the surface. *θ* is the angle of cutting between the cutting holes and the free surface of the cutting, usually 60° ≤ θ ≤ 80°. *β* is the angle of fissure-inducing holes, consider that if this angle is too small, it will cross at the bottom of the hole, and consider the difficulty of construction, 70° ≤ *β* ≤ 90°.

The shear resistance of face C_1_D_1_D_2_C_2_ is:

3 $$Q_{{C_{1} D_{1} D_{2} C_{2} }} = (c + \sigma_{1} \tan \varphi )\left( {d - \frac{L}{\tan \theta }} \right)L$$

The shear resistance of face A1A2C2C1 and face B1B2D2D1 is

4 $$Q_{{A_{1} A_{2} C_{2} C_{1} }} = Q_{{B_{1} B_{2} D_{2} D_{1} }} = ({\text{c}} + \sigma_{2} \tan \varphi )(n - 1)a\frac{L}{\cos \theta }$$

where *σ*_*2*_ is the positive stress acting on its surface, $\sigma_{2} = \frac{\nu }{1 - \nu }\sigma_{1}$, and $\nu$ is the rock’s Poisson ratio.

Face A_2_B_2_C_2_D_2_ tensile resistance is

5 $$T = (n - 1)a\sin \beta \left[ {d - (n - 1)a\cos \beta - 2\frac{L}{\tan \theta }} \right]\sigma_{{\text{t}}}$$

From the above, the total resistance to cutting into a cavity at the line of least resistance *Q* is

6 $$Q = (Q_{{A_{1} A_{2} C_{2} C_{1} }} + Q_{{B_{1} B_{2} D_{2} D_{1} }} )\sin \beta \cdot \sin \theta + Q_{{A_{1} B_{1} B_{2} A_{2} }} + Q_{{C_{1} D_{1} D_{2} C_{2} }} + T$$

**Figure 4:**
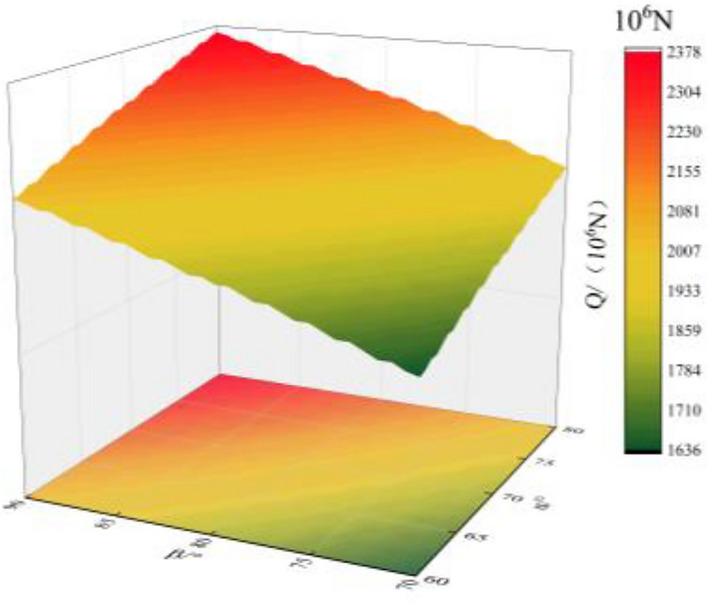
Relationship between cutting angle *θ* and fissure-inducing angle *β* and total resistance *Q.*

The relationship between the cutting angle *θ* and the fissure-inducing angle *β* and the total resistance *Q* is shown in Fig. 4.

From Fig. 4, it can be seen that the angle *θ* between the wedge-shaped cutting holes and the tunnel face, and the fissure-inducing angle *β* of the cutting holes have a great influence on the formation of the cutting cavity. With the increase of the cutting angle *θ*, the shear resistance on the face A_1_B_1_B_2_A_2_ and the face C_1_D_1_D_2_C_2_ increases, which leads to the increase of the total resistance *Q*. As the layup angle *β* increases, the tensile resistance on face A_2_B_2_D_2_C_2_ increases, leading to an increase in the total resistance *Q*. The clamping angle *θ* is related to the shear resistance, while the clamping angle *β* is related to the tensile resistance. However, the clamp angle *θ* has a greater effect on the total resistance *Q*.

The blasting process can be regarded as an isentropic expansion process; according to isentropic expansion, the static pressure of the transmitted shock wave in the rock acting on the wall of the gun-hole is:

7 $$P_{p} = \left( {\frac{{d_{c} }}{{d_{b} }}} \right)^{2n} P$$

where *P*, the burst pressure, is 10GPa. *d*_*b*_, the gun-hole diameter, was 42mm. *n*, the isentropic index, was 3.

A single hole in the slot cavity acting on the wall of the static pressure can be described by:

8 $$P_{L} = P_{P} L_{C} d_{b}$$

where *L*_*C*_ is the length of the gun-hole charge, *L*_*c*_ = 2.4m.

The cutting hole bursting gas static pressure along the direction of the minimum resistance line in the center of the slot cavity of the combined force can be described by:

9 $$F_{1} = NP_{L} \cos \theta$$

where *N* is the total number of cutting holes, 16.

The combined force of the bursting gas in the direction of vertical excavation surface of the inclined gun-hole is:

10 $$F_{2} = NP_{P} \frac{\pi }{4}d_{b}^{2} \sin \theta$$

I In order for the rock in the cutting chamber to be thrown out, it is necessary to satisfy *F*_*1*_ + *F*_*2*_ ≥ *Q* + *T*

11 $$\begin{aligned} & (Q_{AA^{\prime}C^{\prime}C} + Q_{BB^{\prime}D^{\prime}D} )\sin \beta \cdot \sin \theta + Q_{ABB^{\prime}A^{\prime}} + Q_{CDD^{\prime}C^{\prime}} + T \\ & \quad \le NP_{P} (L_{C} d_{b} \cos \theta + \frac{\pi }{4}d_{b}^{2} \sin \theta ) \\ \end{aligned}$$

The calculation results are shown in Table 3.

**Table 3:**
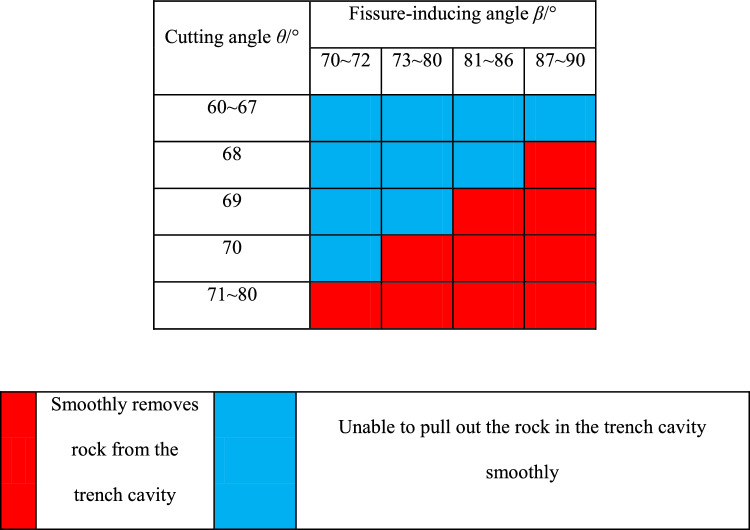
Theoretical calculation results.

As can be seen from Table 3, when the cutting angle is between 60° ≤ *θ* ≤ 70° and 70° ≤ *β* ≤ 74°, the rock body in the slot cavity can be hollowed out smoothly. When the cutting angle is between 60° ≤ *θ* ≤ 69°, 70° ≤ *β* ≤ 78°, the rock can be pulled out smoothly. When the cutting angle is between 60° ≤ *θ* ≤ 68°, 70° ≤ *β* ≤ 86°, the rock can also be pulled out smoothly. When the cutting angle is between 60° ≤ *θ* ≤ 67°, 70° ≤ *β* ≤ 90°, the rock can be pulled out smoothly as well. In the remaining cases,* F*_*1*_ + *F*_*2*_ ≥ *Q* + *T* cannot be satisfied, and the rock in the trench cavity cannot be thrown out, resulting in a failure of trenching.

From the calculation results, it can be seen that the cutting angle *θ* is the main influencing factor. when the cutting angle *θ* is large and the rock cannot be hollowed out smoothly, adjusting the fissure-inducing angle *β* can make the rock body in the slot cavity be hollowed out smoothly by *F*_*1*_ + *F*_*2*_ ≥ *Q* + *T*.

From Eqs. (9), (10) and calculations, when θ ≤ 67°, the difference of total resistance *Q* can be controlled within 10%. When θ ≤ 65°, the combined force *F*_*1*_ + *F*_*2*_ is larger, and the following difficulties are prone to occur: the slot cavity of the crushed rock is too large a distance; it is easy to smash the operating equipment; the pile of slag is too dispersed, which is not conducive to the rapid discharge of slag; and at the same time, the depth of the gun-hole is limited, which is not conducive to large-scale rapid construction. From Table 2, it can be seen that when θ is larger than θ, it is not possible to pull out smoothly. In addition, it can be seen that when θ ≥ 71°, the combined force *F*_*1*_ + *F*_*2*_ is too small, and cutting cannot easily succeed. Therefore, the fissure-inducing angle of wedge-shaped cutting holes should satisfy 68° ≤ θ ≤ 70° and 70° ≤ β ≤ 72°, which is in line with the provision of relevant specifications^24^.

## Numerical simulation

### Finite element model

**Figure 5:**
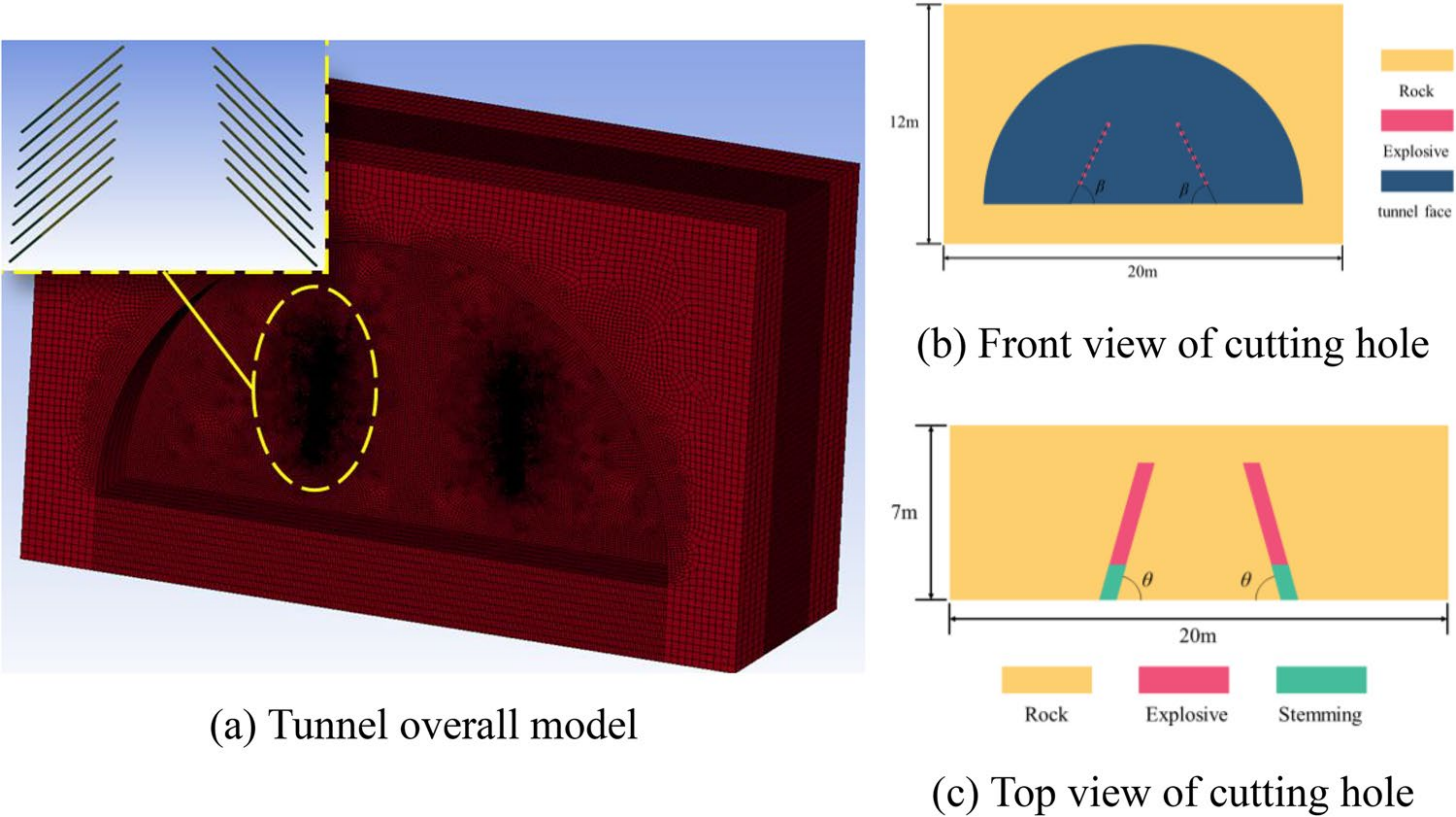
Schematic diagram of the structure of the cutting hole.

ANSYS/LS-DYNA software was used to establish 25 numerical models of wedge-shaped cutting holes with different cutting angles and different fissure-inducing angles with three-dimensional dimensions of 20m × 12m × 9m, all of which used 8-node solid164 units, and the units were taken as g-cm-μs. The spacing of the bottom cutting holes was 5.6m, and the rest of the boundaries were set up with no-reflecting boundaries, except for the palm surface of the tunnel, so as to effectively avoid the influence of reflected waves caused by artificial boundaries on the calculation results. A conventional Lagrange algorithm is used for the rock body, the ALE algorithm was used for air, gun clay and explosives, and the total simulation time was set to 4000 μs. The schematic diagram of the wedge-shaped cutting holes is shown in Fig. 5.

### Material selection

#### Parameter determination

Currently, three damage ontology models, the Holmquist–Johnson–Cook (HJC) model^25^, RHT model^26^ and JH series model^27^, are usually used to simulate rock blasting damage. Compared with other models, the RHT model can effectively describe the tensile and compressive damage evolution of rock under dynamic loading. Therefore, the RHT model was used for subsequent numerical simulations in this paper.

The model parameters were calibrated using test data. A series of physical and mechanical tests were carried out based on the limestone of the Bayue Mountain Tunnel in Chongqing, China, and the test procedure is shown in Fig. 2. The compacted density of limestone *ρ*_*r*_ = 2750 kg·m^−3^, uniaxial compressive strength *f*_*c*_, shear modulus *G*, and uniaxial tensile strength *T* were obtained as shown in Table 1. The remaining model parameters were determined as follows.

(1) Strain rate parameterization.

Strain rate has a significant effect on the strength of the rock mass. In the RHT model, the relationship between strength and strain rate is:

12 $$F_{{\text{r}}} \left( {\dot{\varepsilon }_{{\text{p}}} } \right) = \left\{ {\begin{array}{*{20}l} {\left( {\dot{\varepsilon }_{{\text{p}}} /\dot{\varepsilon }_{0}^{{\text{c}}} } \right)^{{\beta_{{\text{c}}} }} P \ge f_{{\text{c}}} /3} \hfill \\ {\frac{{P + f_{{\text{t}}} /3}}{{f_{{\text{c}}} /3 + f_{{\text{t}}} /3}}\left( {\dot{\varepsilon }_{{\text{p}}} /\dot{\varepsilon }_{0}^{{\text{t}}} } \right)^{{\beta_{{\text{c}}} }} - \frac{{P - f_{{\text{c}}} /3}}{{f_{{\text{c}}} /3 + f_{{\text{t}}} /3}}\left( {\dot{\varepsilon }_{{\text{p}}} /\dot{\varepsilon }_{0}^{{\text{c}}} } \right)^{{\beta_{{\text{t}}} }} - f_{{\text{t}}} /3 < P < f_{{\text{c}}} /3} \hfill \\ {\left( {\dot{\varepsilon }_{{\text{p}}} /\dot{\varepsilon }_{0}^{{\text{t}}} } \right)^{{\beta_{t} }} P \le - f_{{\text{t}}} /3} \hfill \\ \end{array} } \right.$$

where $\dot{\varepsilon }_{0}^{{\text{c}}}$ is the reference strain rate for compression, $\dot{\varepsilon }_{0}^{{\text{c}}}$ = 3.0 × 10^-5^s^−1^. $\dot{\varepsilon }_{0}^{{\text{t}}}$ is the reference strain rate for tension, $\dot{\varepsilon }_{0}^{{\text{t}}}$ = 3.0 × 10^-6^s^−1^. *P* is the pressure. *β*_*c*_ and *β*_*t*_ are the material constants for compression and tension, respectively, *β*_*c*_ = 4/(20 + 3*f*_*c*_) = 0.0117 and *β*_*t*_ = 2/(20 + *f*_*c*_) = 0.0157.

(2) Determination of damage surface parameters.

When 3*P*_0_^*^ ≥ *σ*_f_^*^, the damage surface is expressed as.

13 $$\sigma_{{\text{f}}}^{*} \left( {P_{0}^{*} ,F_{{\text{r}}} } \right) = A\left( {P_{0}^{*} - F_{{\text{r}}} /3 + \left( {A/F_{{\text{r}}} } \right)^{ - 1/N} } \right)^{N} ,3P_{0}^{*} \ge F_{{\text{r}}}$$

where *σ*_f_^*^ is the normalized strength, *σ*_f_^*^ = *σ*_*f*_/*f*_*c*_, *σ*_*f*_ = *σ*_*1*_*-σ*_*3*_. *A* and *N* are the damage surface parameters.

**Figure 6:**
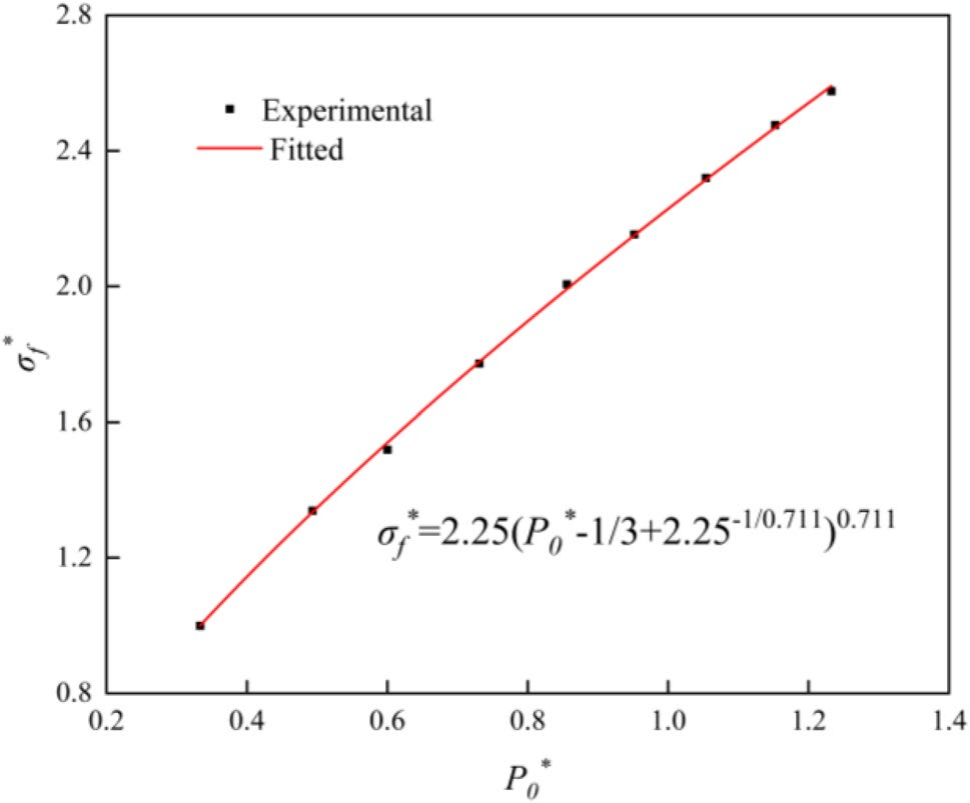
Parameter fitting for damage surface.

The two parameters *A* and *N* can be determined via testing. The triaxial compression test of limestone is shown in Table 3, and P0^*^ and *σ*_f_^*^ can be obtained via calculation. When the material is in a quasi-static state, *F*_*r*_ = 1. According to the data in Table 2, the fitting Eq. (19) determines that *A* = 2.25 and *N* = 0.711, and the fitting results are shown in Fig. 6.

(3) Determination of damage parameters.

In the RHT model, the accumulation of plastic strain *ε*_*p*_ is used to define the damage value *D*

14 $$D = \sum {\frac{{d\varepsilon_{{\text{p}}} }}{{\varepsilon_{{\text{p}}}^{{\text{f}}} }}}$$

where *ε*_*p*_^*f*^ is the plastic strain at damage.

When a given stress state reaches the ultimate strength of the material at the damage surface, damage accumulates in the form of further inelastic deformation or plastic strain. The plastic strain at damage is

15 $$\varepsilon_{{\text{p}}}^{{\text{f}}} = \left\{ {\begin{array}{*{20}l} {D_{1} \left[ {P_{0}^{*} - (1 - D)P_{{\text{t}}}^{*} } \right]^{{D_{2} }} P_{0}^{*} \ge (1 - D)P_{{\text{t}}}^{*} + \left( {\varepsilon_{{\text{p}}}^{{\text{m}}} /D_{1} } \right)^{{1/D_{2} }} } \hfill \\ {\varepsilon_{{\text{p}}}^{{\text{m}}} P_{0}^{*} < (1 - D)P_{{\text{t}}}^{*} + \left( {\varepsilon_{{\text{p}}}^{{\text{m}}} /D_{1} } \right)^{{1/D_{2} }} } \hfill \\ \end{array} } \right.$$

where *ε*_*p*_^*m*^ is the minimum damage residual strain, *P*_t_^*^ is the pressure at failure, and *D*_*1*_and *D*_*2*_ are damage constants, taken as *D*_1_ = 0.04 and *D*_2_ = 1.0.

(4) Determination of the parameters of the equation of state of the rock mass *p*-*α.*

In the RHT model, the equation of state for the *p*-*α* compaction of the rock mass is

16 $$P_{R} = \frac{1}{{\alpha_{0} }}((B_{0} + B_{1} \mu )\alpha_{0} \rho_{0} e + A_{1} \mu + A_{2} \mu^{2} + A_{3} \mu^{3} )$$

where *P*_*R*_ is the pressure of the equation of state, *α*_*0*_ is the initial porosity, *B*_*0*_ and *B*_*1*_ are the material constants, *B*_*0*_ = *B*_*1*_ = 1.68, *e* is the internal energy per unit mass, *μ* is the volumetric strain, and *A*_*1*_, *A*_*2*_ and *A*_*3*_ are the polynomial coefficients, which are given by

17 $$A_{1} = \alpha_{0} \rho_{0} c_{0}^{2} = T_{1}$$

18 $$A_{2} = \alpha_{0} \rho_{0} c_{0}^{2} (2k - 1)$$

19 $$A_{3} = \alpha_{0} \rho_{0} c_{0}^{2} (3k^{2} - 4k + 1)$$

where *c*_*0*_ is the speed of sound at room temperature and pressure, *T*_*1*_ is the material constant, and k is the empirical constant of the material. *T*_*1*_, *A*_*1*_, *A*_*2*_ and *A*_*3*_ are 45.62 GPa, 45.62 GPa, 76.64 GPa, and 46.84 GPa, respectively. *P*_*crush*_ is the crushing pressure, *P*_*crush*_ = 2fc/3^28^, and is taken as *P*_*crush*_ = 71.35MPa. *G*_t_^*^ is the yield surface parameter, taken as *G*_t_^*^ = 0.7.

(5) Parameter tuning and optimization.

**Figure 7:**
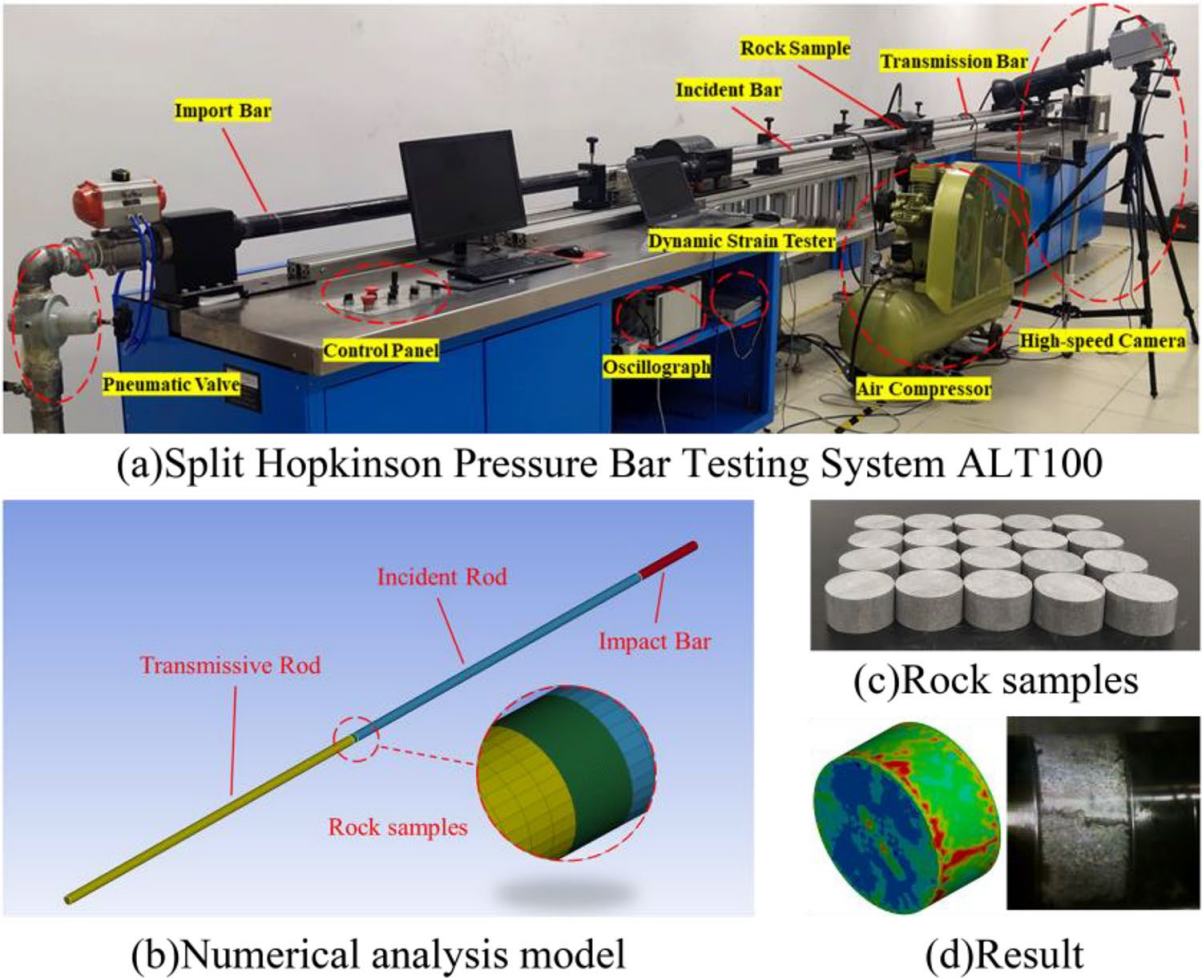
SHPB test.

The remaining parameters are difficult to determine, but Borrvall and Riede^26^ recommended their reference values. The parameters that are sensitive to numerical results include the yield surface parameter *G*_c_^*^, the residual surface parameters *A*_*f*_ and *N*_*f*_, and the relative shear strength f^*^. They were adjusted and optimized according to the specific test results. Other parameters that are not sensitive to the simulation results were directly quoted from Borrvall and Riedel^26^. A series of dynamic compression tests were conducted on limestone specimens using the split Hopkinson pressure bar (SHPB) test system^29^. These remaining parameters can be determined via a numerical simulation of these dynamic splitting tests. The numerical model of the limestone specimen is shown in Fig. 7. For the SHPB system, the lengths of the incident and transmission rods were 2000 mm and 2000 mm, respectively. The diameter of the specimen was 50 mm and the height was 25 mm. The model dimensions were exactly the same as in the tests. The damage pattern of the specimen at an impact velocity of 20.4m·s^−1^ is given in Fig. 7d, and the numerical results are basically consistent with those of the indoor tests. The results show that the RHT model can describe the damage range and dynamic rupture of the rock samples more accurately. The fully determined parameters are detailed in Table 4.

**Table 4:** Parameters of RHT model for limestone.

Parameter	Value	Parameter	Value	Parameter	Value
*ρ*_*r*_ (kg·m^-3^)	2645	*F*_t_*	0.03	*P*_*crush*_(MPa)	71.35
*G* (GPa)	7.97	*F*_s_*	0.35	*G*_c_*	2.0
*f*_*c*_ (MPa)	107.02	*A*_1_ (GPa)	45.62	*G*_t_*	0.7
*N*	0.711	*A*_2_ (GPa)	41.06	*XI*	0.5
*β*_*t*_	0.0157	*A*_3_ (GPa)	-4.22	*D*_1_	0.04
*B*_*0*_	0.9	*Q*_0_	0.6805	*D*_2_	1.0
*B*_*1*_	0.9	*B*	0.0105	*β*_*c*_	0.0117
*α*_*0*_	1.04	*EOC* (s^−1^)	3.0E-5	*A*_*f*_	1.57
*T*_1_ (GPa)	45.62	*EOT* (s^−1^)	3.0E-6	*N*_*f*_	0.58
*P*_*lock*_ (GPa)	6	*A*	2.25	*N*_*p*_	3

#### Explosive material

No. 2 emulsion explosives were used in the field, and MAT_ HIGH_ EXPLOSIVE_ BURN material was selected^30^), which was combined with the JWJ equation of state to describe the relationship between bursting pressure and specific volume in the process of artillery bombardment^31^.

20 $$P_{{\text{J}}} = A_{{\text{J}}} \left( {1 - \omega /R_{1} V} \right)e^{{ - R_{1} v}} + B_{{\text{J}}} \left( {1 - \omega /R_{2} V} \right)e^{{ - R_{2} v}} + \omega E_{J} /V$$

where *P*_*J*_ is the burst pressure. *V* is the volume. *E*_*J*_ is the initial internal energy. *A*_*J*_, *B*_*J*_, *R*_*1*_, *R*_*2*_, *ω* are the parameters of JWL equation of state. The explosive material density is 1240 kg·m^−3^. The burst speed is 4200 m·s^−1^, Poisson's ratio is 0.33, and the burst pressure *P*_*J*_ = 1.3GPa.

#### Stemming material

MAT_SOIL_AND_FOAM material was selected for the plugging^30^, with a material density of 1900 kg·m^−3^, a modulus of elasticity of 3.3 GPa, a Poisson ratio of 0.33, and a yield strength of 0.65 MPa.

#### Air material

Air, as a fluid material, was selected as MAT_NULL^30^, the equation of state was selected as the LINEAR_POYNOMIAL equation, and the relevant parameters are *C*_*1*_ = *C*_*2*_ = *C*_*3*_ = 0,*C*_*4*_ = *C*_*5*_ = 0.4,*E*_*0*_ = 0.25MPa, *V*_*0*_ = 1, where *E*_*0*_ is the initial internal energy and *V*_*0*_ is the initial volume.

## Analysis of numerical calculation results

### Effective stress wave analysis

**Figure 8:**
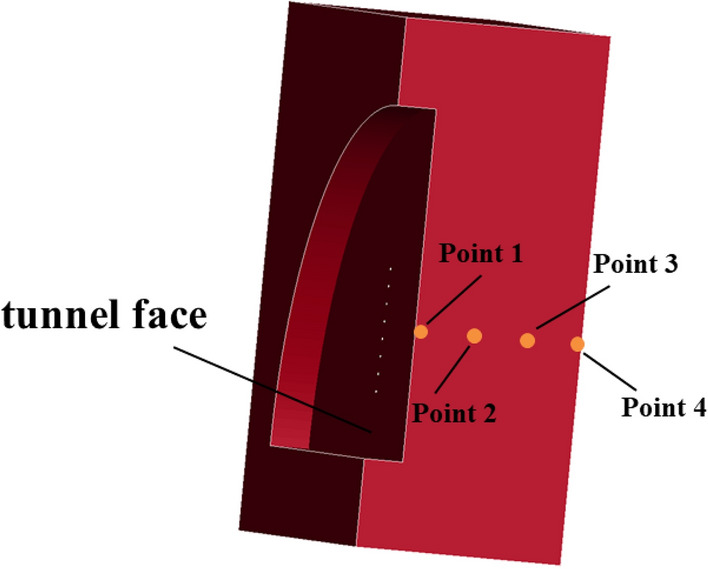
Schematic diagram of the location of selected units.

In order to analyze the five different cutting angles (60°, 65°, 70°, 75°, 80°) and five different fissure-inducing angles (70°, 75°, 80°, 85°, 90°) in the damage process of the size of the effective stress, the establishment of the numerical model of 25 different potentialities for III enclosing rock was necessary. We created 25 models of the symmetry surface from the palm face 0 cm, 100 cm, 200 cm, and 300 cm from the same unit using the effective stress data, as shown in Fig. 8., which depicts 25 types of hole deployment after detonation in the form of ellipsoidal propagation, as well as the phenomena of reflected stress waves and positive stress wave superposition when the propagation reaches the free face of the stress wave reflection.

**Figure 9:**
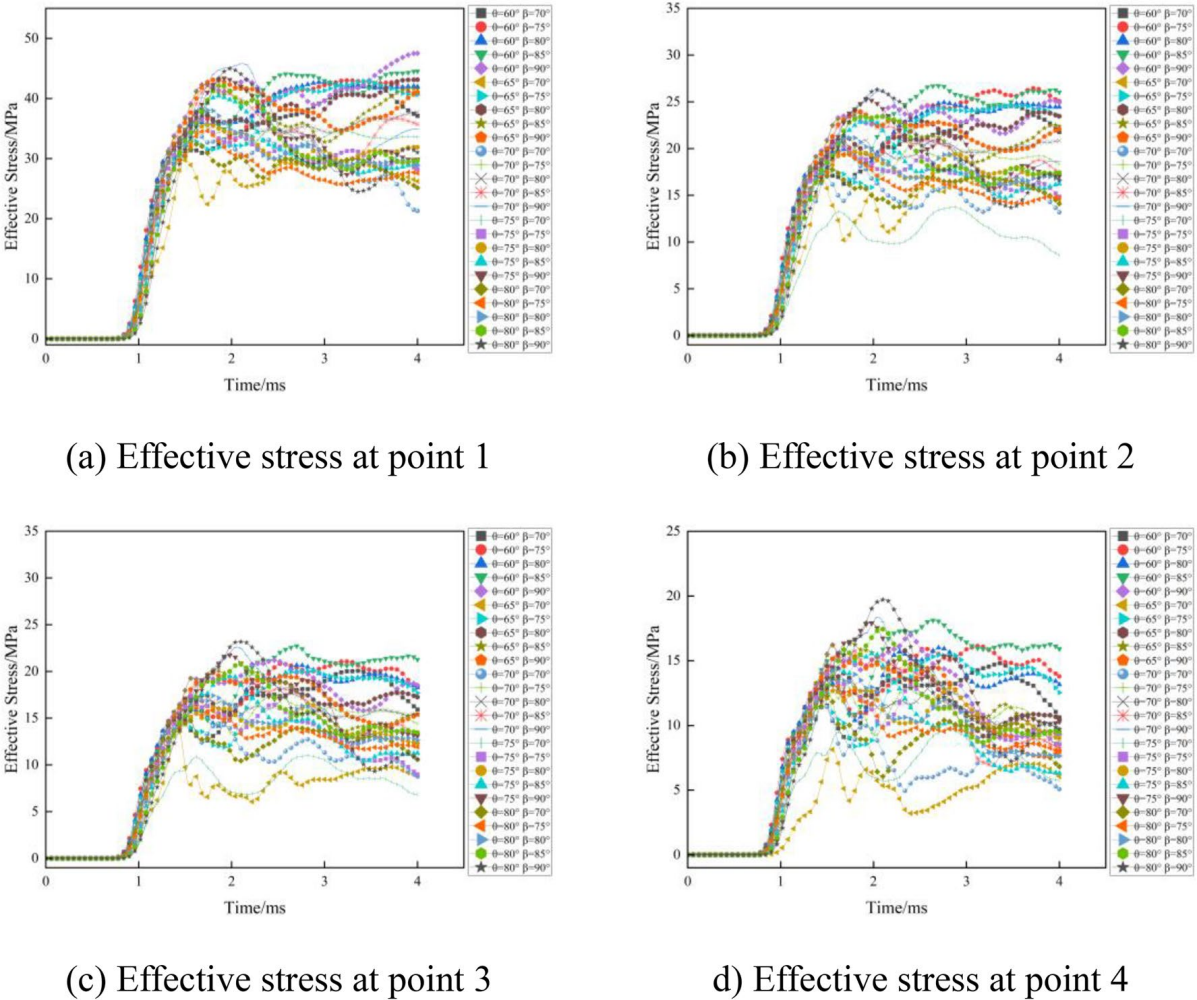
Effective stresses for different deployment forms.

As can be seen from Fig. 9, when the fissure-inducing angle in the same location of the effective stress is very different from the cutting angle, the effective stress gradually decreases with increasing cutting angle, while the effective stress increases with deployment angle. At point 1, due to the superposition of the stress wave, the effective stress is larger. At point 4, the effective stress is smaller because of the superposition of the instant explosion of the stress wave, causing rock damage and consuming a large amount of energy, resulting in a reduction in the effective stress.

The smaller the fissure-inducing angle of the cutting holes, the more energy is consumed in rock destruction during blasting, the better the cutting effect. In the case of a constant cutting angle of the cutting holes, appropriately reducing the fissure-inducing angle of the cutting holes and maximizing the performance of the explosives to make use of the rock in the cutting area makes it more easily broken. The effect of the cutting is better, and the results of the theoretical calculations are in line with the results.

### Dynamic damage of surrounding rock

**Figure 10:**
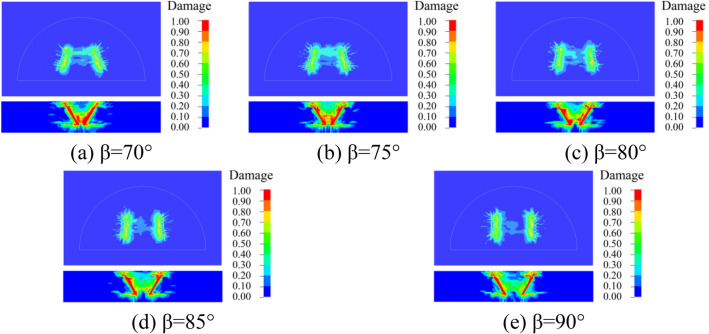
Damage for different unfolding angle *β* deployment methods when *θ* = 60°

**Figure 11:**
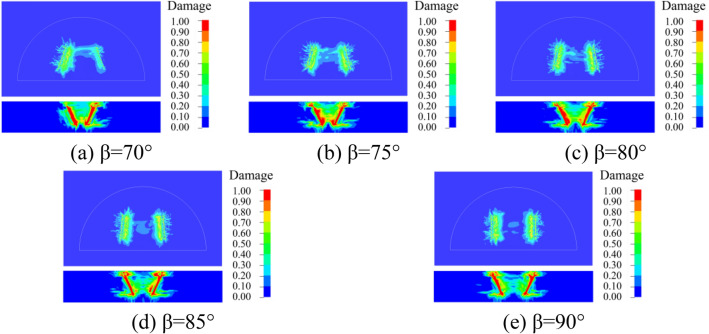
Damage for different unfolding angle *β* deployment methods when *θ* = 65°

**Figure 12:**
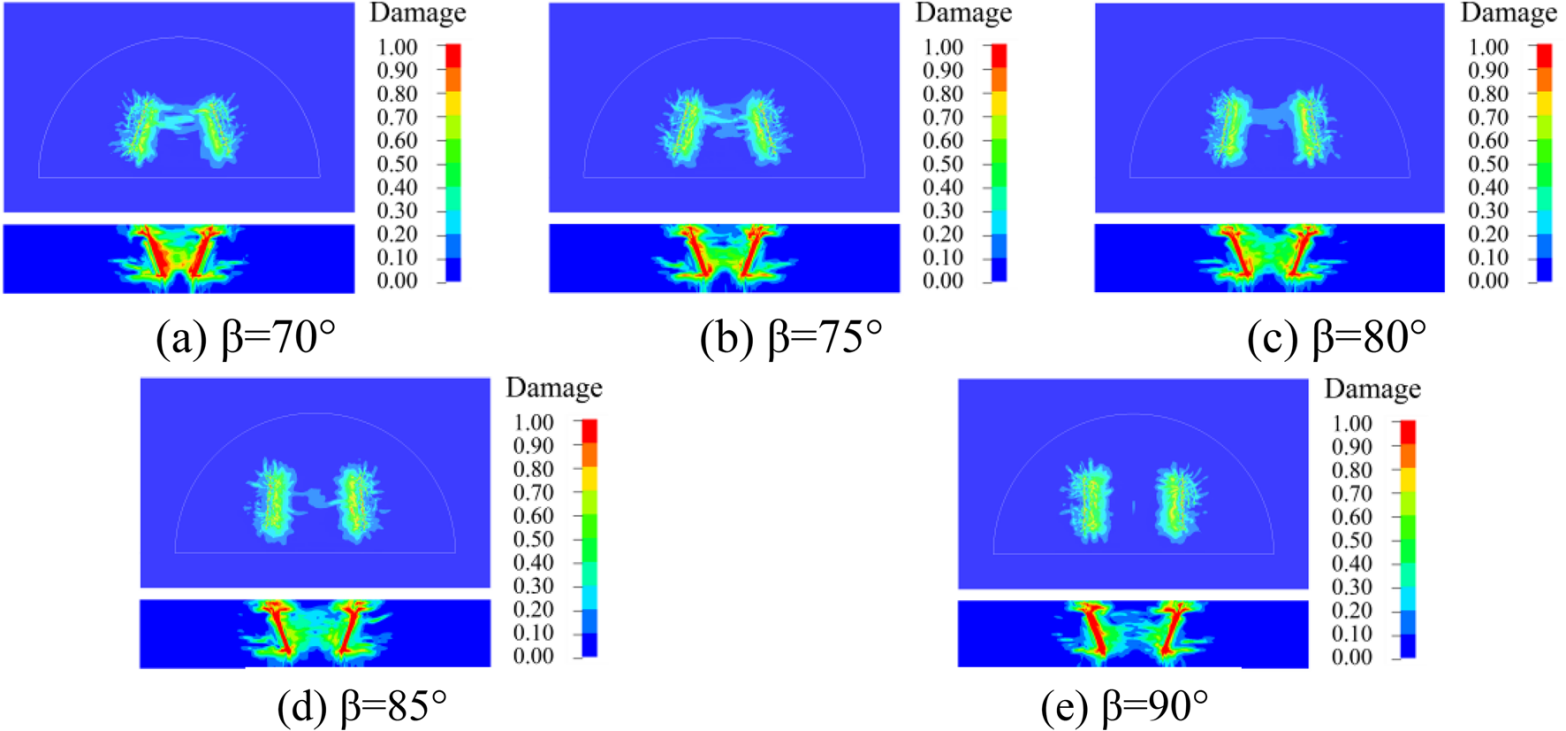
Damage for different unfolding angle *β* deployment methods when *θ* = 70°

**Figure 13:**
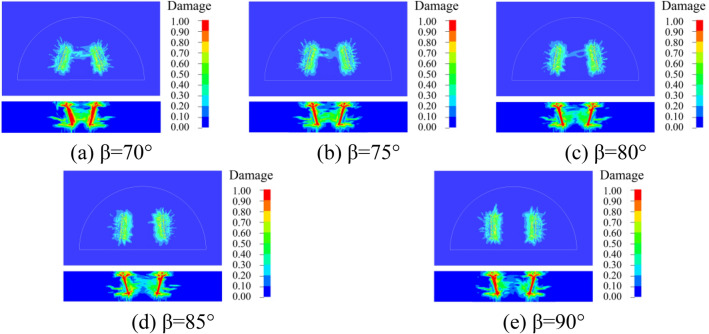
Damage for different unfolding angle *β* deployment methods when *θ* = 75°

**Figure 14:**
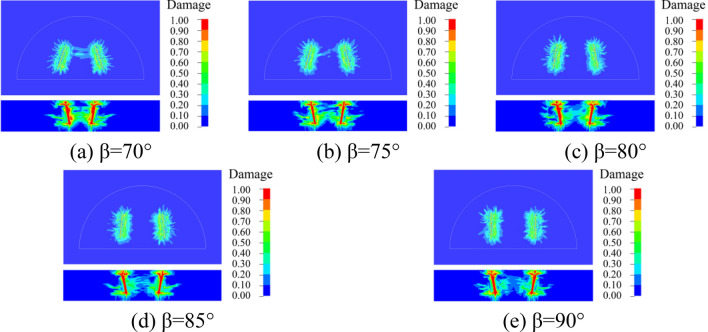
Damage for different unfolding angle *β* deployment methods when *θ* = 80°

In order to better understand the damage of the surrounding rock with different cutting and fissure-inducing angles of the wedge-shaped cutting holes, an analysis was carried out by using the damage criterion that comes with the RHT model, which was defined as 4#history. The damage of the 25 different deployment modes of the holes is shown in Figs. 10, 11, 12, 13 and 14.

As shown in the figure, the damage areas of the 25 types of hole deployment methods are different, but all of them have a wider range of damage along the axial sides of the gun-hole; the damage at the mouth of the gun-hole is smaller, and the damage to the surrounding rock at the bottom of the gun-hole is larger. When the cutting angle is 60°, the damage area at the bottom of the gun-hole is the flattest, eliminating the "bulging belly" phenomenon, and providing a more regular palm surface of the new tunnel. When the cutting angle is 65°, the damage area at the bottom of the hole is relatively flat, with no "bulging belly" phenomenon, and the damage range of the surrounding rock is slightly larger than the cutting angle of 60°. When the cutting angle is 70°, the damage area at the bottom of the hole is relatively flat, and the damage range of the surrounding rock is slightly larger than that at the cutting angle of 65°, with a slight " bulging belly " phenomenon at the bottom of the hole, which was within the acceptable range. When the cutting angle is 75° and 80°, the damage area of the surrounding rock is larger, but the distance between the bottom of the holes is too large, resulting in the rock body between the holes being not broken, and the whole damage area is the smallest, which means that it is necessary to increase the rate of explosives consumption to achieve the same effect.

As shown in Figs. 10, 11, 12, 13 and 14, when the cutting angles are 60°, 65° and 70°, the influence of the change in fissure-inducing angle on the damage range of the surrounding rock is negligible, and the use of larger fissure-inducing angles can effectively expand the damage range of the surrounding rock. When the cutting angle is 75° and 80°, the damage range of the surrounding rock increases with the decrease in the fissure-inducing angle *β*. Therefore, when the cutting angle is large, using a smaller fissure-inducing angle can effectively improve the blasting effect without changing the explosive unit consumption rate.

## Method validation

In order to further verify the applicability of the wedge-shaped cutting hole placement method in large-cross-section tunnels, in this paper, we carried out on-site application studies in the Bayue Mountain Tunne.

Bayue Mountain Tunnel is located near Shiyu Town, Tongliang District, Chongqing, China. The total length of the tunnel is 2701 m, with a maximum burial depth of 311 m. The perimeter rock of the on-site test section (K12 + 250 ~ K12 + 300) is of level III perimeter rock, the lithology is mainly limestone, and the perimeter rock at the test section is relatively stable and is constructed by the upper and lower step method, with the upper step excavation area of about 114 m^2^.

**Figure 15:**
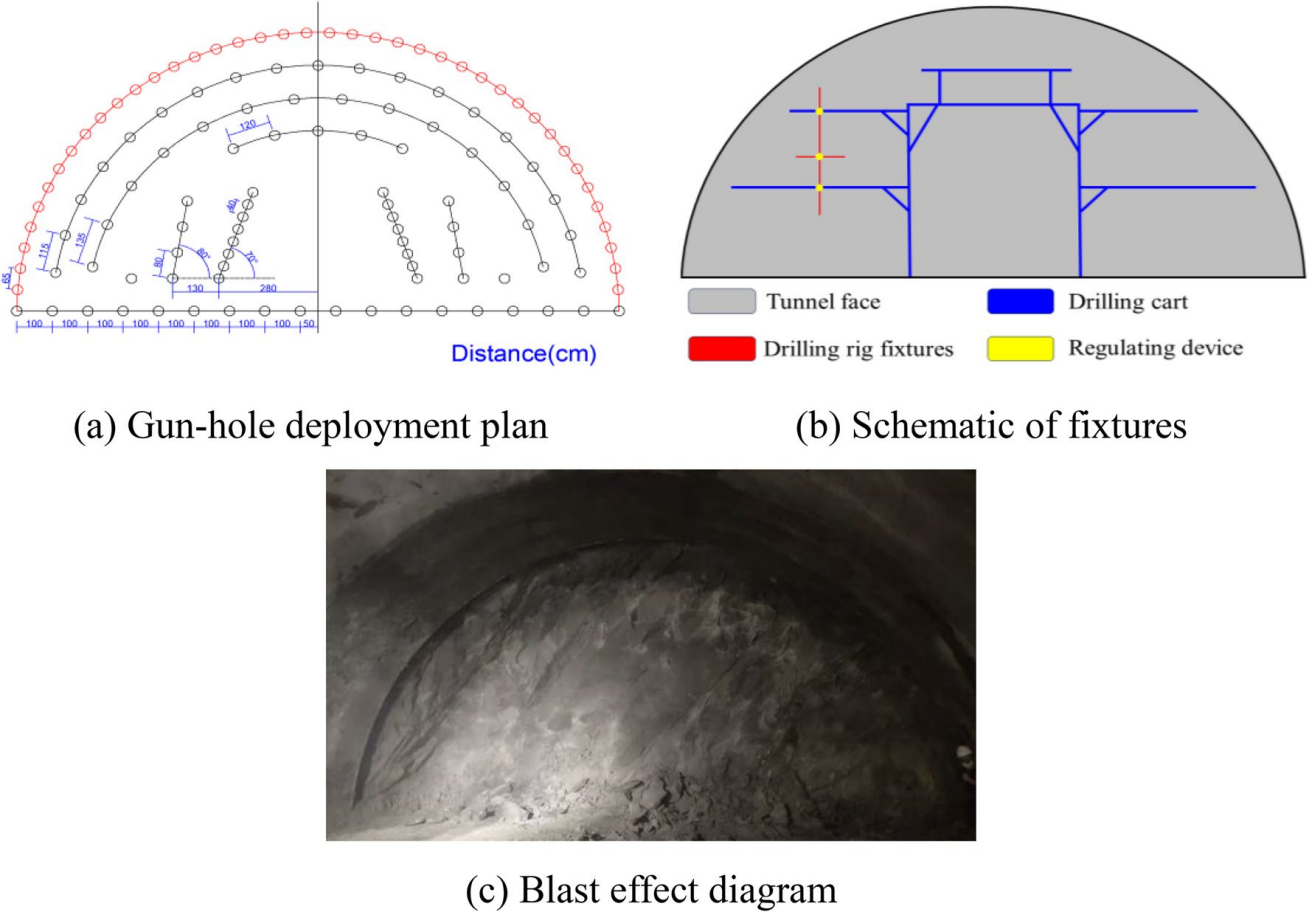
The deployment of the holes and the effect after blasting.

In order to ensure the accuracy of the gun-hole layout, in this on-site test, the position of the gun-hole on the tunnel face was firstly measured with a measuring tape and marked using spray paint with a measurement error of ≤ 1cm, and the gun-hole layout diagram is shown in Fig. 15a. Drilling was carried out via manual drilling, using rock drilling rods with a length of 4m and a drill bit with a diameter of 42mm, with a cutting angle and a fissure-inducing angle of 70°, and the explosives used were No. 2 emulsion explosives, with the specification of the medicine roll being φ32mm*300mm*300g. For the initiator, we used a millisecond delay electronic digital detonator with a high-power generator. In order to avoid manual drilling caused by the deviation in angle of the cutting hole and avoid potentially affecting blasting, the construction cart was modified to add an adjustable drilling rig fixture so that the manual drilling angle could be controlled in the range of ± 1°; the fixture’s schematic diagram is shown in Fig. 15b. Adopting this hole arrangement, we carried out 10 blasting tests in the Bayue Mountain Tunnel, and then the actual footage after blasting and the utilization rate of the holes were counted, because the overall blasting effect of the tunnel depends on the cutting out blasting, so the overall blasting effect index was used to indirectly evaluate the effect of the cutting out blasting. The effect after blasting is shown in Fig. 15c, and the statistics of the blasting effect are shown in Table 5.

**Table 5:** Gun-hole utilization rate.

Number	Design feed distance/m	Actual feed distance/m		Gun-hole utilization rate/%		Is there a " bulging belly "?	
		Original	Modify	Original	Modify	Original	Modify
1	3	2.48	2.79	82.67	93.00	yes	No
2			2.74		91.33		No
3			2.79		93.00		No
4			2.78		92.67		No
5			2.77		92.33		No
6			2.80		93.33		No
7			2.74		91.33		No
8			2.77		92.33		No
9			2.79		93.00		No
10			2.82		94.00		No
Average			2.779		92.63		

As can be seen from Table 5, the designed footage of the Bayue Mountain Tunnel is 3m. After using the hole layout method proposed in this article, the average actual footage distance increased from 2.48 to 2.779 m, and the utilization rate of blast holes increased from 82.67 to 92.63%, an increase of nearly 10%. At the same time, there was no occurrence of "bulging belly". The blasting effect is good, verifying that the cutting holes proposed in this paper have applicability to the fissure-inducing of holes.

## Discussion

In order to verify the generalizability of the method proposed in this paper, 10 blasting tests were conducted in the Gonghe Village Tunnel of the Ludian–Qiaojia Expressway in Yunnan, China.

**Figure 16:**
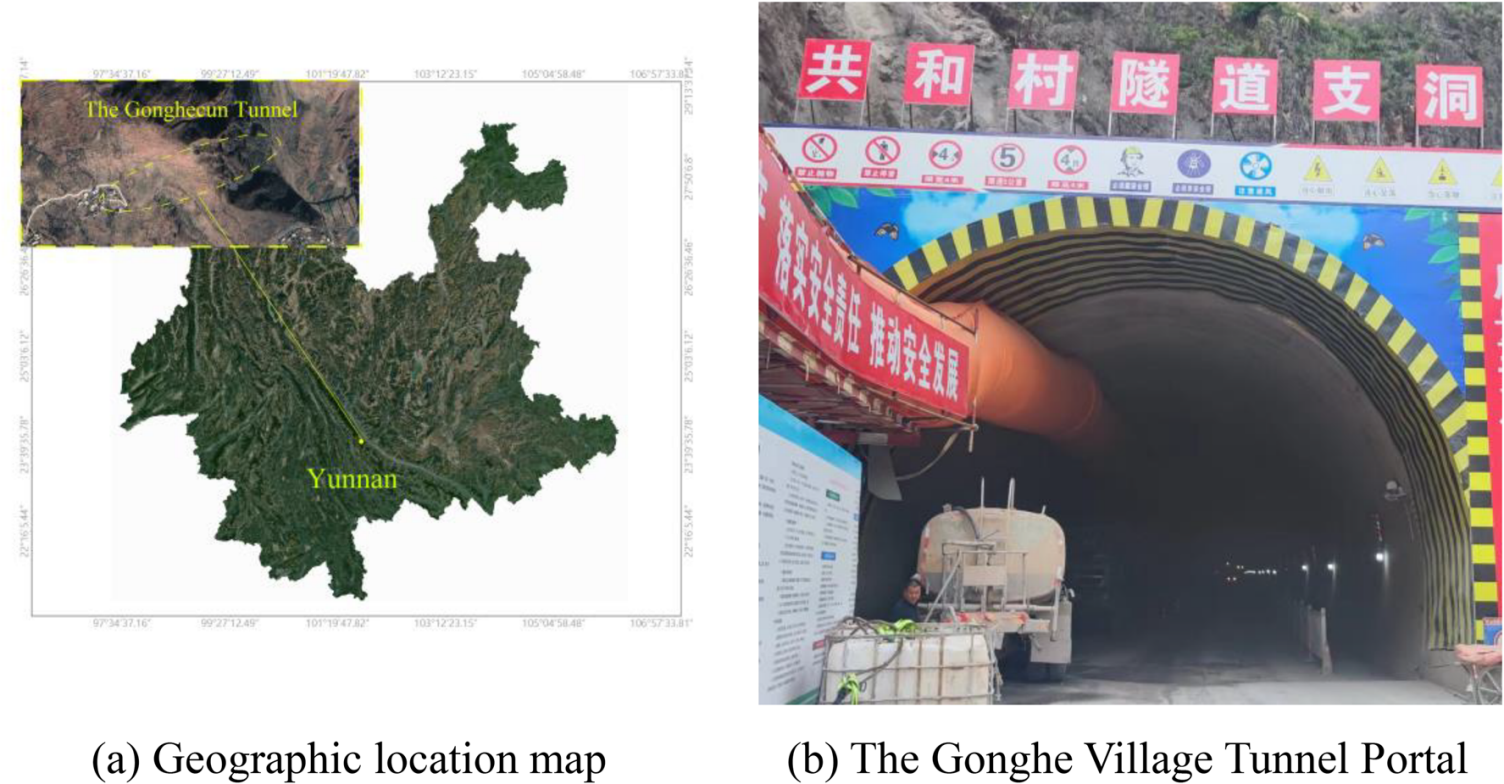
The Gonghe Village Tunnel.

The Gonghe Village Tunnel is a split tunnel with a curved spread. The total length of the tunnel is 10,734 m, with a maximum burial depth of 1322 m. Upper and lower step blasting were used, and the upper step excavation area was about 85m^2^, as shown in Fig. 16. The surrounding rock of the on-site test section (K46 + 410 ~ K46 + 510) is level III, the lithology is mainly limestone.

**Figure 17:**
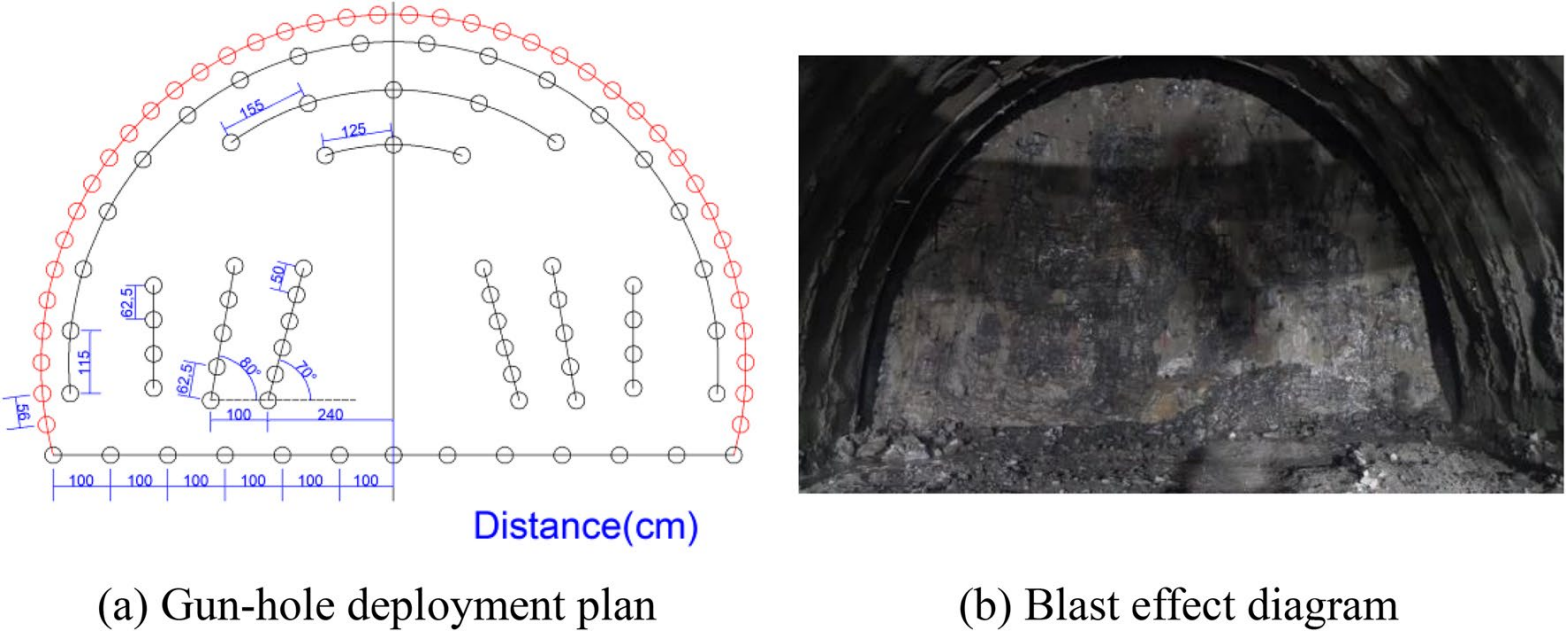
The Gonghe Village Tunnel gun-hole deployment and Post-Blast Effects.

The testing was carried out using the hole placement method proposed in this paper, and the cutting angle and fissure-inducing angle were both 70°, as shown in Fig. 17a. The effect after blasting is shown in Fig. 17b, and the statistics of blasting effect are shown in Table 6.

**Table 6:** The Gonghe village tunnel gun-hole utilization rate.

Number	Design feed distance/m	Actual feed distance/m		Gun-hole utilization rate/%		Is there a " bulging belly "?	
		Original	Modify	Original	Modify	Original	Modify
1	3.5	2.73	3.23	78.00	92.29	Yes	No
2			3.25		92.86		No
3			3.41		97.43		No
4			3.33		95.14		No
5			3.35		95.71		No
6			3.27		93.43		No
7			3.38		96.57		No
8			3.38		96.57		No
9			3.25		92.86		No
10			3.35		95.71		No
Average			3.32		94.86		

As can be seen from Table 6, the designed footage of the Gonghe Village Tunnel is 3.5 m. After using the hole layout method proposed in this article, the average actual footage distance increased from 2.73 to 3.32 m, and the utilization rate of blast holes increased from 78.00 to 94.86%, an increase of over 15%. At the same time, there was no occurrence of "bulging belly", the blasting effect is good.

In summary, the use of the wedge-shaped cutting hole proposed in this study has good generalizability and is suitable for other large section third level surrounding rock limestone tunnels.

## Conclusion

In this paper, an efficient and refined blasting method for wedge-shaped cutting holes in large-cross-section tunnels is proposed with Bayue Mountain Tunnel as the engineering background. The influence of different forms of hole placement on the blasting effect was studied, and theoretical analysis, numerical simulation and on-site testing were carried out. The following conclusions can be obtained.

1. Changing the cutting angle *θ* changes the shear resistance on face A_2_B_2_D_2_C_2_ in the three-dimensional model of wedge-shaped cutting, so the total resistance *Q* to cutting into a cavity increases with the increase in cutting angle *θ*. Changing the fissure-inducing angle *β* changes the shear resistance on face A_1_B_1_B_2_A_2_ and face C_1_D_1_D_2_C_2_, so the total resistance *Q* to cutting into a cavity increases with fissure-inducing angle *β*.
2. After theoretical derivation, it was found that for level III surrounding rock, under the condition that the cutting angle of the cutting holes remains unchanged, the fissure-inducing angle of the cutting holes ought to be appropriately reduced to maximize the performance of the explosives so as to make the rock in the cutting area easier to break and create a better cutting effect.
3. Through numerical simulation, it was found that when the cutting angle is *θ* ≤ 65°, there is no "bulging belly" phenomenon; when the cutting angle is *θ* = 70°, the bottom of the gun-hole has a slight "bulging belly" phenomenon; and when the cutting angle is 75° and 80°, the surrounding rock damage is larger, but the area between the bottom of the gun-holes is larger. When the cutting angle is 75° and 80°, the surrounding rock damage area is larger, but the spacing between the bottom of the holes is too large, resulting in the rock body between the holes being not broken, and the whole damage area is the smallest, which means that to achieve the same blasting effect, it is necessary to increase the rate of explosives consumption.
4. For level III surrounding rock, the angle of wedge-shaped cutting holes should meet 68° ≤ θ ≤ 70° and 70° ≤ β ≤ 72°. With the method proposed in this paper, on-site tests were carried out in the Bayue Mountain Tunnel and the Gonghe Village Tunnel, both of which were successfully trenched, and the average utilization rates of the gun-holes were effectively improved to 92.68% and 94.86%, respectively.

## Data Availability

The datasets used during the current study available from the corresponding author on reasonable request.
